# Neural correlates of body size estimation: A systematic review and narrative synthesis

**DOI:** 10.1016/j.bbr.2025.116020

**Published:** 2025-12-30

**Authors:** Hayden J. Peel, Akansha M. Naraindas, Joyce Guo, Valentina Cazzato, Jamie D. Feusner

**Affiliations:** a Centre for Addiction and Mental Health, Toronto, ON, Canada; b La Trobe University, Melbourne, Victoria, Australia; c Centre for Public Health, Queens University Belfast, United Kingdom; d University of California San Diego, San Diego, CA, USA; e Department of Cognitive Sciences, Psychology, Education, and Cultural Studies, University of Messina, Messina, Italy; f Faculty of Health, Liverpool John Moores University, Liverpool, United Kingdom; g Department of Psychiatry, Division of Neurosciences & Clinical Translation, University of Toronto, Toronto, ON, Canada; h Department of Women’s and Children’s Health, Karolinska Institutet, Stockholm, Sweden

**Keywords:** Body image perception, Systematic review, Narrative synthesis, FMRI

## Abstract

Inaccurate body size estimation (BSE), the discrepancy between an individual’s actual and perceived body size and shape, is observed not only in clinical conditions like eating disorders and body dysmorphic disorder but also in healthy individuals. Understanding the neural mechanisms that support BSE is timely, given growing interest in perceptual biases and their potential relevance for identifying mechanisms that may be disrupted in clinical populations. However, the field has an incomplete understanding of brain systems functionally involved in BSE ability. To address this, we performed a systematic review, accompanied by a narrative synthesis, to identify brain regions associated with BSE across studies of healthy individuals. Studies using functional neuroimaging were selected if they elicited BSE with a task, contrasted BSE with a control task, and used whole-brain analyses (rather than being restricted to a priori regions of interest). This yielded a set of nine functional magnetic resonance imaging papers. There is relatively consistent involvement of ventral (fusiform/occipitotemporal regions) and dorsal (intraparietal areas) visual pathways, and discrete regions of the prefrontal cortex, suggesting recurring engagement of perceptual and higher-order cognitive systems during BSE. However, current knowledge is limited by the small number and heterogeneity of available studies. We identify both consistent and variable neural correlates of BSE, offering refined targets for future investigations of BSE in clinical populations. Based on these findings, we additionally provide specific suggestions for improving neuroimaging task design for future studies.

## 1. Introduction

Body image is a multidimensional construct encompassing perceptions and attitudes toward one’s body, primarily regarding, but not limited to, physical appearance [1]. The attitudinal dimension relates to an individual’s feelings of satisfaction or dissatisfaction with their body size and shape [2]. Perceptual body image, which is conceptually linked to how accurately individuals estimate their own body size and shape, may be a function of neural systems that might encompass a wide range of processes, including visual, somatosensory, proprioceptive, vestibular, multisensory, and mental imagery networks [3].

Perceptual body image is often assessed through body size estimation (BSE) tasks, which evaluate an individual’s ability to judge the size and shape of their body [2,4,5]. BSE tasks are heterogenous and typically rely on either metric or depictive techniques [6]. Metric methods require participants to estimate their body size using spatial tools (e.g., calipers, tape measures), whereas depictive methods, more commonly used in neuroimaging research, involve comparing one’s body to another stimulus such as an avatar [7], or adjust digitally altered self-images. In this review, we define BSE in line with Molbert et al.’s [6] classification of BSE including both depictive and metric tasks given the diversity of experimental designs in the current neuroimaging literature.

Inaccurate BSE can lead to body dissatisfaction, reduced self-esteem, and adverse psychological and physical health outcomes, including depression, anxiety, and excessive exercise [8]. Furthermore, errors in BSE can contribute to body image disturbances in psychiatric conditions. Body size distortions, often manifesting as overestimation of body size, are particularly pronounced in anorexia nervosa [9,10] and body dysmorphic disorder [11]. However, non-clinical participants also misestimate their body size [9,12–14]. For example, Cornelissen et al. [15], demonstrated that both individuals with anorexia nervosa and healthy participants show systematic perceptual errors when estimating their own body size using computer-adjusted images. Specifically, underweight healthy participants, similar to those with anorexia, tended to overestimate their body size, whereas higher-BMI healthy participants tended to underestimate it. This pattern is ascribed to the phenomenon of contraction bias, a perceptual tendency in which judgments are pulled toward an internal reference point: stimuli smaller than the reference are overestimated, while larger stimuli are underestimated [16]. Such inaccuracies could potentially influencing health behaviors across clinical and non-clinical populations. For instance, underestimation of one’s body size can diminish motivation for weight loss in obese and overweight individuals [17]. Conversely, individuals who overestimate their size are more likely to engage in unhealthy dieting behaviours [18]. Despite these wide-ranging consequences, the neural correlates and key systems underlying BSE, outside a clinical context, remain poorly understood.

Previous reviews have attempted to synthesise aspects of body representation, but these efforts remain limited in several ways. First, progress in the field is hindered by inconsistent terminology and unclear taxonomies, making it difficult to compare findings across studies. Second, much of the existing literature focuses predominantly on clinical groups, which constrains our understanding of body processing to deficit-based models and provides insight primarily from individuals already diagnosed with body image disturbances [19]. Yet the mechanisms underlying body representation e.g. such as multisensory integration, interoception, and visual-perceptual processing operate across all individuals. Investigating BSE in the general population is therefore essential for establishing baseline functioning and understanding the natural variability of these processes. Some reviews have sought to distinguish between the attitudinal and perceptual components of body image, which have found, for example, selective associations between body size misestimation and functional alterations in the precuneus and inferior parietal lobe in individuals with anorexia [20]. In contrast, other reviews have combined attitudinal and perceptual dimensions, conceptualising little difference between these constructs [21]. Recent reviews have moved beyond taxonomic classifications of body representation, towards a more functional classification, demonstrating that body size misestimations occur in both static and dynamic body representations [22]. Notably, a meta-analysis conducted in healthy populations has provided evidence of distinct functional activation patterns for both action-oriented (i.e., body schema: a sensorimotor construct integrating afferent and efferent information to guide movement), and non-action-oriented (visual-spatial imagery-based) body representations [23]. The latter, which is more closely aligned with performing BSE, was found to selectively activate the primary somatosensory cortex (S1) and supramarginal gyrus (SMG), further supporting a neural distinction between these representational systems [23]. However, no review to date has specifically synthesized the neural correlates of BSE in healthy individuals.

Previous fMRI studies in healthy participants have identified key brain areas involved in processing body images, including the extrastriate body area (EBA) [24], fusiform body area (FBA) [25], S1 [26], and temporoparietal junction (TPJ) [27], with additional evidence implicating parietal regions such as the intraparietal sulcus (IPS) [28] and the left postcentral sulcus [29]. However, variability in stimuli, task paradigms, and participant populations across studies limits the generalizability of findings from each study, especially from small samples [30]. Further, while these regions may be broadly implicated in body processing, it remains unclear how they contribute specifically to BSE. Therefore, a systematic review focused on the neural correlates of BSE in non-clinical populations is needed to establish potential neurobiological mechanistic features in the absence of clinical pathology to assess if, and how, and to what extent, different clinical samples deviate from typical patterns.

In this study, we aimed to identify patterns of functional brain activity associated with BSE in healthy populations by synthesizing data from existing fMRI studies. We hypothesized that regions previously implicated in both body processing and self-referential processing, that is, the processing of those stimuli that are experienced as strongly related to one’s own person, would demonstrate significant activation during BSE tasks. Specifically, we predicted recruitment of regions in the ventral (EBA, FBA) and dorsal (IPS, inferior parietal and superior parietal lobule [IPL, SPL]) visual streams, reflecting the visual demands of BSE tasks, with the former providing body-selective visual processing [22,23] and the latter contributing spatial [31,32] and feature-based visual processing [33]. Additionally, we predicted activation in the right TPJ, given its role in processing visual information about one’s own body and its spatial position [34,35], as well as S1, which supports body ownership and BSE [36]. Finally, we predicted involvement of the medial prefrontal cortex (mPFC), a region integral to self-referential thinking [37], since BSE tasks inherently involve thinking about how another stimulus relates to oneself. To test these hypotheses, we conducted a systematic review followed by a qualitative narrative synthesis to understand the brain regions that underlie own-body size estimation. Furthermore, because BSE tasks vary considerably across studies, we also aim to map and synthesise the different task types employed in these paradigms.

## 2. Methods

### 2.1. Design

This systematic review was conducted and reported in accordance with the PRISMA guidelines [38]. The study protocol was also registered on PROSPERO (CRD42024620548).

### 2.2. Literature search and selection criteria

The research question and search strategy for this review were formulated using the PCC framework (Population, Concept, Context). The search strategy was created using MeSH (Medical subject heading) terms and keywords derived from the following 1) Population: Individuals in the general population, 2) Concept: Body size estimation, 3) Context: the studies searched for in the review were specifically neuroimaging studies. As an example, the search strategy for the PubMed database was as follows: Search keywords included (“body image” OR “body image distortion” OR “body size estimation” OR “body shape” OR “body thickness”) AND (“functional magnetic resonance imaging” OR “functional neuroimaging” OR “functional MRI” OR “fMRI”) AND (“eating disorders” OR “anorexia nervosa” OR “bulimia nervosa” OR “body dysmorphic disorder”). A full list of search strings for each database is provided in the Supplementary Methods 1. Only English-language studies were included, and no date limits were applied. We conducted a literature search across PubMed, Web of Science, and EBSCO | PsychINFO to identify relevant studies.

Studies were included if they [1] were carried out using fMRI^2^; [2] were original articles adopting BSE task-related paradigms (i.e., both depictive and/or metric); [3] recruited healthy adult participants 18 years or older^3^; [4] at least part of the analyses performed whole-brain analyses (as opposed to solely region of interest (ROI)-based analyses, which would be limited to, and potentially biased towards, a priori hypothesized regions); [5] reported coordinates of significant clusters in Montreal Neurological Institute (MNI) or Talairach space. Studies were excluded if they [1] recruited participants < 18 years old; [2] were unpublished (e.g., conference abstracts, trial protocols); [3] did not report standard space coordinates, or, we were not able to obtain these coordinates from the authors; [4] only performed ROI analyses; [5] were not published in English. Although this review focuses on BSE in healthy adults, clinical terms (e.g., eating disorders, body dysmorphic disorder) were included in the search strategy because most neuroimaging studies on BSE are conducted with clinical samples that also include healthy control groups. This ensured that all eligible datasets involving healthy participants were captured. During screening, studies involving clinical populations were retained for full-text review to determine whether within-group BSE data were available for healthy controls. Studies reporting only between-group comparisons without extractable within-group data for healthy participants were excluded. We also manually reviewed the reference lists of retrieved articles to identify additional studies. The first search was conducted in April 2024 and repeated in June 2025 with no other additional studies identified for inclusion.

### 2.3. Study selection

Fig. 1 provides an overview of all databases and identified references. The search produced 263 studies and was imported to Endnote X9 for deduplication. No time window was specified for the search. Once duplicates were removed, 78 studies were uploaded to the Covidence review management software for title and abstract screening. Two authors (A.M.N., J.G) performed 100 % double title and abstract, and full-text screening independently (See Fig. 1). Inter-rater agreement at full-text screening was moderate (Cohen’s Kappa = 0.45) [39]. We had an arbitration plan in place whereby a third reviewer (JF) would adjudicate any disagreements unresolved by the two primary reviewers. JF would independently assess the material and join a discussion to reach a final decision. In practice, all discrepancies were resolved through consensus between the primary reviewers, and third-reviewer arbitration was not required. Following this process, 44 studies were included for full-text review and 35 were excluded. The full list of studies excluded at the full-text stage, along with reasons for exclusion, is provided in Supplementary Methods 2. Overall, a total of 9 studies were included in the review and 100 % data extraction was completed by AMN.

### 2.4. Data extraction

A data extraction template was designed in accordance with the Cochrane guidelines [40] and tailored to meet the specific objectives of this study. The extracted data from eligible studies encompassed: [1] study characteristics, including publication year, country and study design; [2] participant demographics, such as sample size, age, and gender distribution; and [3] task-related details, including the type of BSE task and the control task utilized. Additionally, information on t-thresholds, data analysis software, neuroimaging coordinates (x, y, z), and corresponding effect sizes (p, t, or z) were extracted. The full data extraction is located in Supplementary Table S2.

### 2.5. MRI quality assessment

The quality of included studies was assessed according to published guidelines for standardized reporting of MRI studies [41,42]. All studies reported their general MRI design, including software packages used, image acquisition parameters, pre-processing procedures, and first/second-level analyses where applicable. All studies reported magnetic field strength, which was either 1.5 T (6/9) or 3 T (3/9), with scanners including Philips (2/9), Siemens (3/9), General Electric (3/9), and Magnex Eclipse (1/9).

### 2.6. Data synthesis

A narrative synthesis approach was employed to summarize the extracted data [43]. This approach involves a textual summary and interpretation of the main findings from studies included in the systematic review. Whilst an Activation Likelihood Estimation (ALE) meta-analysis was initially planned to synthesise the results of the review, the final number of included studies was insufficient to meet recommended thresholds for statistical power, in line with best practice guidelines for conducting ALE analyses which recommend a minimum of 17 studies [44]. Therefore, an exploratory ALE meta-analysis was conducted and the results are presented in Supplementary Table S2 and Figure S1. As with all narrative syntheses, the interpretation of findings is limited by methodological heterogeneity across studies, including variation in BSE paradigms and imaging pipelines.

## 3. Results

### 3.1. Sample characteristics

Nine studies were deemed eligible according to the inclusion criteria, consisting of 174 healthy adult participants (Table 1). The studies included in this review were conducted in Japan (*n* = 4), China (*n* = 1), USA (*n* = 2), UK (*n* = 1) and Italy (*n* = 1). Among these, five presented participants with distorted images of their own body, whereas the other four required participants to compare their own body to others. The control task varied between studies, including house viewing tasks [2], viewing their own or another body stimulus (usually in those where distorted images were presented) [2], shape size judgements [1], or presenting scrambled body pictures [3].

### 3.2. Neuroimaging findings

The imaging findings are listed in Table 2, followed by a narrative synthesis of regions described across studies.

#### 3.2.1. Reported regions: similarities and differences

Across the nine studies, consistent engagement of prefrontal, ventral visual, and parietal regions was observed, although the specific loci varied across paradigms. Prefrontal engagement was commonly reported, with multiple studies identifying activity in the middle frontal gyrus (MFG), inferior frontal gyrus (IFG), and dorsolateral prefrontal cortex (DLPFC) [45–49,52]. DLPFC activations were predominantly right-lateralized [46,47,52], although bilateral involvement was noted in Miyake et al. [47]. The mPFC was noted in several studies [45,48,49, 52], including bilateral engagement in Gao et al. [52].

Ventral visual stream regions, including fusiform gyrus, middle/inferior occipital gyrus, lingual gyrus, and inferior temporal gyrus, were consistently reported [45–47,50,52]. These activations were typically bilateral, though some studies reported right-lateralized clusters [45, 52]. Dorsal / parietal visual areas, including the inferior and superior parietal lobules (IPL/SPL) were reported in four studies [46,47,50,52], primarily right-lateralized [46,50,52], though bilateral involvement was also observed [50,52].

Several regions were less frequently or singly reported. This included the amygdala in three studies [45,47,52], and the parahippocampal gyrus in four [46,47,50,52]. The anterior cingulate cortex (ACC) [27, 46], superior temporal gyrus (STG) [45,51], premotor cortex [47,48] precentral gyrus [48], putamen [47], and precuneus and cuneus [48,50, 52] were also detected infrequently. The right TPJ was only reported in one study [27], as with the secondary somatosensory cortex (S2) [51].

#### 3.2.2. BSE Tasks used across studies: similarities and differences

In this review, five studies presented distorted own-body photographs [27,45,47,50,51], and four required participants to mentally compare their body to another visually presented body stimulus [46,48, 49,52]. Of the five studies that presented distorted own-body photographs, there were notable differences in the paradigms and tasks participants performed. Two papers [45,47] used identical paradigms, where own-body photographs were distorted to appear larger or smaller, and participants were instructed to select the more subjectively ‘unpleasant’ image when presented with a set of altered images alongside the original. Similarly, another [51] presented distorted own-body photographs in both horizontal and dorsoventral orientations, but the task required participants to judge whether the images depicted their own body or someone else’s. Another study [27] also created distorted images, but used silhouette versions of participants’ bodies instead of full-feature photographs (this distinction is noteworthy given that BSE errors are more pronounced when using silhouettes compared to full-feature body images [53]). Such differences in stimuli limit the ability to generalize the findings across these nine studies. The fifth study in this group [50] presented distorted own-body photographs in a passive viewing paradigm, where participants were not explicitly tasked with judging whether the images differed from their own body. In contrast, the remaining four studies tasked participants with comparing their own body size to other-body stimuli. Three of these [48,49,52] used the same set of 42 virtual avatars, depicting “thin” (aligned with societal ideals) and “fat” (exceeding societal norms) body types. Participants were instructed to imagine someone comparing their body to the one depicted in the image (e.g., “Imagine someone is saying, ‘your body looks like hers’”) [49]. In contrast, the final paper [46] presented participants with images of slim female bodies taken from women’s magazines, instructing them to compare their own body shape with those in the images. Overall, whilst these tasks varied in the stimuli used (although three studies used the same set of stimuli), focus, and instructions, they nevertheless all probed participants’ perceptions of their own body size and shape.

## 4. Discussion

This study aimed to identify consistent patterns of functional brain activity associated with BSE in healthy individuals by synthesising existing fMRI studies. Accurate BSE is a key component of body image, and understanding its neural basis in healthy populations is important for determining how these systems may be disrupted in conditions like eating disorders [6,9,21] and body dysmorphic disorder [11] However, the neural correlates of BSE in healthy populations remain poorly understood. To address this gap, we performed a systematic review of fMRI-based BSE tasks in healthy participants. Across several studies, we found evidence of engagement during BSE tasks, often bilaterally, of the ventral visual pathway (FBA and EBA), dorsal visual pathway (IPL/SPL), and prefrontal cortex (DLPFC, IFG, MFG, mPFC). These findings are largely consistent with hypothesized regions. However, the TPJ and S1 were infrequently or singly reported.

### 4.1. Extrastriate and fusiform body areas

Among the most robust findings in the neuroscience of body perception, is the engagement of regions in the ventral visual stream that appear to be ‘body-selective’: the EBA and fusiform body area FBA [24, 25]. While the EBA, situated laterally and posteriorly, is sensitive to body parts and visual features, the more ventral and anterior FBA is associated with holistic body representations and more invariant processing [54–56]. All studies included in this review employed whole-body stimuli, and five reported fusiform gyrus activation consistent with FBA engagement [45–47,50,52], while three reported occipitotemporal or inferior occipital activation consistent with EBA [45,50,52]. Notably, three studies [45,50,52] reported patterns consistent with co-activation of both regions, suggesting joint recruitment of local and configural visual processing mechanisms during BSE.

FBA involvement was more consistent across the studies included in the review, appearing in tasks that required participants to evaluate distorted body images [45,47], make affective or comparative judgments [46,52], or passively view distorted own-body photographs [50]. Several studies that contrasted body stimuli with non-body stimuli—such as houses [50], interior design images [46], or scrambled bodies [52]—reported robust FBA activation. These findings suggest that the FBA is more strongly engaged by images depicting intact human bodies relative to control stimuli lacking coherent body structure or biological relevance, supporting its sensitivity to whole-body forms. This likely accounts for the findings of Owens et al. [48] and Spangler and Allen [49], who, despite using the same distorted avatar stimuli as Gao et al. [52], did not report activation in the FBA. This potentially could be due to using within-body contrasts (fat versus thin; thin versus fat), rather than contrasts between body and non-body stimuli. Such contrasts may elicit subtler perceptual differences than those observed when comparing bodies to non-body stimuli, thus limiting FBA recruitment. More broadly, this pattern suggests that the FBA may not be strongly recruited during BSE tasks where body form is always present and the perceptual distinction lies in relative size or shape—indicating that BSE may rely more on regions beyond canonical body-selective areas when explicit body detection is not required.

Similarly, Hamamoto et al. [27], who used silhouette images of participants’ bodies contrasted with shapes, did not find FBA involvement. This may reflect the reduced configurational, surface feature, and depth cues in silhouettes. The authors reasoned that silhouette images would provide substantial anonymity and potentially encourage participation. However, silhouettes, despite maintaining the global configuration of a body, might be somewhat closer perceptually to shapes than are photographs of holistic body stimuli, reducing the specificity derived from this contrast. Notably, a separate study using body silhouettes reported FBA, EBA, and lateral occipital complex engagement [57], suggesting that earlier visual areas involved in shape and outline processing may also contribute to the neural representation of body silhouettes. Thus, the level of visual detail and the nature of the contrast can influence the extent to which body-selective areas are recruited. Finally, Hashimoto et al. [51] (although using body photographs) reported a cluster in the left fusiform gyrus consistent with FBA, but it did not survive correction for multiple comparisons, suggesting that sensitivity rather than true absence may explain the non-significant result. Together, these findings suggest that FBA is recruited when visual body stimuli are configural and richly featured (as is the case in photographs), and contrasted with non-body control stimuli. Overall, as activation of the FBA was reported in six of the nine studies reviewed (including one non-significant result), it was the most frequently implicated region in BSE tasks identified in this review.

EBA activation was identified in only three studies—Kurosaki et al. [45], Castellini et al. [50], and Gao et al. [52]—despite the use of comparable body stimuli and task contrast across several other studies. In Castellini et al. [50] and Gao et al. [52], participants viewed distorted body stimuli contrasted with non-body images (e.g., houses or scrambled bodies), suggesting that EBA may be particularly responsive to the presence of intact body structure in such contrasts. However, not all body versus non-body contrasts elicited EBA engagement. For instance, Hamamoto et al. [27] did not report EBA activation when contrasting body silhouettes with geometric shapes, nor did Friederich et al. [46], who contrasted body images with interior design scenes. A more striking discrepancy emerges between Kurosaki et al. [45] and Miyake et al. [47], both of which employed nearly identical within-body contrast designs, yet only Kurosaki et al. [45] reported EBA activation. Both studies were likely underpowered (both with n = 11). Further the absences of significant EBA activation may reflect unaccounted-for individual differences in visual processing, such as a focus on local features versus global shape, which could influence EBA recruitment and contribute to variation, which could be more of an issue for a signal to emerge in a small sample. None of the included studies explicitly instructed participants to adopt a part-based or holistic viewing strategy, leaving such factors unmeasured. These findings suggest that while the EBA is sensitive to body parts and local shape, its involvement in BSE tasks is less consistent than the FBA. This variability may stem from factors like contrast structure, stimulus quality, attention, and overlap with adjacent regions. Larger studies or meta-analytic approaches such as an ALE (once more studies have been conducted) are needed, although current evidence is too limited to support an analysis focused on this region.

### 4.2. Dorsal / parietal visual areas

Dorsal parietal regions—including the inferior and superior parietal lobules (IPL, SPL)—were reported in four of the nine reviewed studies [46,47,50,52], predominantly right-lateralized [46,47,50], with bilateral engagement observed in Castellini et al. [50] and Gao et al. [52]. Although IPL/SPL activations were not ubiquitous, their presence in nearly half the studies could potentially reflect the involvement of body-based mental imagery processes, and in particular visuospatial imagery, in BSE [22]. Tasks in these studies varied but shared common demands on spatial comparison. For example, Gao et al. and Friederich et al. [46,52] both required participants to mentally compare their own body to another figure (either an avatar or a slim model) which likely necessitated mapping between internal and external spatial representations. Similarly, Miyake et al. [47] involved judging distorted own-body images, a task that may recruit body-based spatial imagery to make BSE judgements. Interestingly, Castellini et al. [50] observed bilateral IPL/SPL activation even during passive viewing of distorted own-body images, suggesting that recruiting own body visuospatial imagery may occur spontaneously in response to distorted self-representations.

These findings are consistent with existing theories of dorsal stream involvement in both action-oriented and perceptual tasks. While traditionally associated with visuomotor control [58,59], the dorsal stream—including IPL and IPS—also supports perceptual encoding of object-centered spatial relations [60]. In the context of BSE, these regions may supply a spatial scaffold for evaluating size, proportion, and shape, working in concert with ventral visual areas such as the FBA. The observation that IPL/SPL activation was not significant in all studies may reflect, for example, task variability (e.g. if explicit body comparisons were required) or insufficient power. Nevertheless, its emergence across four studies suggests the potential importance of dorsal parietal regions, perhaps subserving spatial body representation in BSE tasks.

### 4.3. Prefrontal involvement in BSE

Medial prefrontal cortex involvement was a key hypothesized region in this review, based on its established role in self-referential processing and affective evaluation in the domain of body image [20,37,61]. Four studies reported mPFC activation [43,46,47,50], including bilateral mPFC in Gao et al. [50]’s overweight group and dorsomedial peaks in Spangler and Allen [49] and Owens et al. [48]. (In the latter, coordinates in the medial superior frontal gyrus ([–10, 56, 29]; [13,24,47]) are anatomically consistent with dorsal mPFC, despite being labeled as superior frontal areas [61].) Interestingly, mPFC activation was not confined to avatar-based or explicitly evaluative paradigms; Kurosaki et al. [45] reported mPFC involvement during an unpleasantness judgment task using distorted own-body photographs. However, mPFC engagement was absent in several other studies using what would seem to be similarly self-relevant stimuli [27,47,50]. This may be due to differences in task framing (e.g., affective evaluation vs. objective discrimination), stimuli (e.g., full-body photos vs. silhouettes), or the extent to which participants were prompted to adopt an introspective or socially comparative stance. For instance, tasks requiring self-other comparisons or imagined social evaluation may more reliably trigger mPFC recruitment than passive viewing or identity discrimination tasks [62]. Additionally, none of the studies distinguished between dorsal and ventral subregions, which may be functionally dissociable [63]. Together, these findings suggest that mPFC engagement during BSE reflects more than just stimulus content; it may depend on how the self is brought into relation with the body, which could be a function of task instructions but may also be different for different participants depending on inherent tendencies for self-referential thinking.

Several studies reported activation in lateral prefrontal regions, including the IFG, MFG, and DLPFC [45–49,52]. Functionally, the MFG has been identified as a key node mediating interactions between the dorsal and ventral attention networks (DAN and VAN), acting as a ‘circuit breaker’ that interrupts ongoing top-down processes to reorient attention toward salient stimuli [31,63,64]. In the context of BSE tasks, this mechanism may facilitate flexible shifts between internally held body representations and visually distorted external input. The right IFG, similarly, has been implicated in interference resolution and mismatch detection [65,66], and is associated with regulating the entry of conflicting perceptual information into conscious awareness [67]. In tasks involving avatar or distorted stimuli, IFG activation may reflect heightened conflict between one’s internal body schema and the manipulated representation, potentially recruiting action observation and motor resonance systems via the Action Observation Network (AON) [68–70]. In line with this, the IFG is consistently engaged when individuals observe body-related stimuli and automatically activate sensorimotor representations associated with those observed body parts [70–72]. Finally, although several studies referred to DLPFC engagement, reported coordinates often overlapped with more functionally specific regions such as the IFG or MFG—underscoring the limitations of broad anatomical labels in the absence of finer-grained parcellation. Taken together, these lateral prefrontal activations may support conflict resolution, evaluative comparison, and attentional control during BSE tasks.

### 4.4. Expected regions that were infrequently reported across studies

The TPJ and S1 were hypothesized to be involved in BSE, but were not consistently reported across studies. Both regions are known to support multisensory integration and body ownership, with the TPJ integrating visual, proprioceptive, and vestibular signals to support self-location and self–other discrimination [34,35,73,74]. It is also commonly engaged during perspective-taking tasks that require embodied mental rotation [75,76]. Most BSE tasks in this review focused on the visual evaluation of body stimuli, which may reduce the demand for spatial self–other comparisons that typically elicit robust TPJ activation. Indeed, only one study [27] reported TPJ involvement, in a task that required participants to judge how well a distorted silhouette aligned with their ideal body shape. Although avatar-based BSE tasks included a comparative element, they were framed more as social-evaluative judgments (e.g., imagining someone commenting on one’s appearance) rather than requiring participants to adopt a third-person spatial perspective. Prior work suggests that TPJ engagement is more reliably observed in tasks requiring explicit perspective-taking or self–location shifts within imagined space [73–76]. Thus, while self–other comparison was conceptually present in several paradigms, the absence of strong embodied perspective transformation may explain the lack of consistent TPJ activation.

Similarly, while S1 is thought to contribute to higher-order body representations beyond simple tactile input [77]—and stimulation of S1 can induce body size estimation distortions for specific body parts [36]—only one study [51] reported secondary somatosensory cortex engagement. This may reflect the purely visual nature of the tasks included in the current review, which did not involve tactile stimulation or bodily interaction. Although somatosensory inputs are central to maintaining and updating internal body maps [78], the visual whole-body images used in most tasks may not have sufficiently engaged somatosensory representations. Furthermore, S1 appears to be more strongly recruited during tasks involving localized body part processing [79], which differs from the whole-body stimuli employed across the reviewed studies.

### 4.5. Methodological and theoretical developments in BSE

Body size estimation, while primarily engaging the perceptual domain of body image, may also be influenced by cognitive, affective, and attitudinal factors, even in healthy individuals [7], reflecting the multidimensional nature of the construct. Our findings contribute to this still somewhat nascent framework by highlighting two primary points: [1] the need for task consistency and precision in measuring BSE, and [2] the proposal that BSE may represent a unique construct, conceptually and functionally distinct from other body image domains. In doing so, our synthesis extends prior meta-analytic and review work on body representation by isolating depictive BSE tasks in healthy individuals, rather than combining broader imagery or linguistic paradigms and clinical samples.

#### 4.5.1. Importance of task consistency and understanding body part processing

We intended this review to broadly assess all types of BSE tasks. In doing so, due to the diverse task demands across the different BSE variations included, there was considerable heterogeneity in the types of stimuli and task instructions used across the studies. Behavioral research has demonstrated that variations in the visual attributes of a body stimulus, such as whether it is presented as a silhouette or with filled-in features [53], its orientation [80], the presence of 3D cues [81], and color [82], can significantly impact errors in BSE.

However, these methodological variations raise an important question: are distorted own-body photographs or other-body stimuli, such as avatars, better suited for probing BSE? Own body photograph-based stimuli are generally considered to have greater ecological validity than avatars, as they invoke self-body recognition (i.e. the ability to distinguish one’s own body from others) [83]. The functional use of this body representation is vital for social interaction and bodily self-awareness, potentially enhancing BSE accuracy [84]. Indeed, behavioral research supports a ‘self-body advantage,’ where individuals respond faster and more accurately to own-body stimuli compared to others [85] (an effect linked to body image concerns for specific body regions [86]). However, importantly, distorted photographs may reduce this advantage [87] due to their limited occurrence in everyday experiences. That is, they are not ecologically valid.

On the other hand, avatar stimuli, despite lower ecological validity than own-body images, maintain strong construct validity by activating mental representations during BSE tasks [88]. Moreover, avatar-based tasks allow greater control and precision in manipulating body part size and shape, revealing region-specific distortions [9,89]. This can offer insights into region-specific body alteration behaviors that may motivate unhealthy dieting and cosmetic surgery [90,91]. Additionally, recent advances in 3D modeling enable real-time avatar adjustments, offering more precise measurement of BSE through manual participant manipulation [9,11,89]. Future research should integrate these technological innovations with functional neuroimaging to identify neural correlates of BSE. Standardizing task parameters and adopting precise, region-specific assessments will enhance generalizability and further elucidate the neural circuits underpinning cognitive and perceptual aspects of BSE.

#### 4.5.2. BSE as a unique construct: conceptual and functional distinctions

While the BSE task is often grouped under the broader category of general perceptual body image measures, our results suggest that own body BSE represents a distinct construct, both conceptually and functionally. Specifically, it appears to differ from earlier conceptualisations of ‘body estimation’ (which combine tasks involving visual stimuli and size-related language, as in [21]), as well as from non-action-oriented forms of body processing (as described in [23]). Prior meta-analyses on body image have included various tasks that capture elements of body estimation. For instance, Di Vita et al. [23] reported a clear neural segregation between body representations involved in action and those that are non-action oriented. According to this framework, BSE, as conceptualized in the present work, would be classified with non-action-oriented body representations. Their analysis revealed that non-action-oriented representations primarily engaged regions such as S1 and the SMG, whereas action-oriented representations involved the primary motor area and right EBA. Of these reported areas, we only found EBA engagement in three studies [45,50,52], none reported SMG (although three reported IPL activity [46,47,52], which may include SMG), and none reported S1, with only secondary somatosensory cortex being reported once [51]. A clear difference in included tasks likely accounts for this discrepancy, as the studies included in Di Vita et al.’s [23] analysis spanned a range of body-based visual imagery tasks, but only one depictive BSE task.

Additionally, BSE paradigms are only as robust as their task instructions. The conflicting instructions across studies included in this review represent a limitation, as they may affect the consistency and comparability of the findings. For example, some studies instructed participants to compare their own body size to underweight or overweight avatars (Gao et al. [52]; Spangler & Allen [49]; Owens et al. [48]), or to idealised body models specifically selected to elicit anxiety during self-comparison [46]. Body specific self-comparison is known to invoke body dissatisfaction [92], which could subsequently influence the accuracy of the participants’ body size estimation. Indeed, BSE tasks are often criticised for the tendency of individuals to rely on attitudinal judgments about their bodies, rather than on visual mental representations, when estimating body size [7,93–96].

Currently, the field is highly conflicted in its definition of BSE, contributing to ambiguity regarding its neural mechanisms. A review by Norrlin and Baumann [21] conceptualized body estimation broadly, including linguistic and imagery tasks, observing increased activation in regions like the middle temporal gyrus and SMG in clinical populations. While experimental studies demonstrate that areas involved in body perception or motor function are also activated when processing related linguistic concepts (e.g. [97–99]), it is crucial to consider how these varied accounts influence how BSE is conceptualized in research. Our review focused exclusively on depictive BSE tasks in healthy individuals, revealing neural activation associated specifically with body processing. Consistent with Molbert et al. [6], an effective measure of depictive BSE must include explicit instructions requiring participants to compare the presented stimuli, whether “fat,” “thin,” or distorted body images, to their own body and make a direct judgment about body size. Without this self-referential component, the task may primarily reflect general body perception rather than the specific cognitive mechanisms underlying BSE. Clarifying these conceptual and functional boundaries is important not only for theory, but also for designing mechanistically precise tasks that can be used to track or modify BSE distortions in at-risk or clinical populations.

### 4.6. Limitations and future directions

Some limitations of the current review warrant consideration. First, the heterogeneity in BSE task paradigms—such as the use of distorted own-body photographs versus virtual avatar comparisons—may have contributed to variability in activation patterns. Moreover, the modest number of experiments [9], with predominantly small sample sizes (average = 15) meant that coordinate-based ALE analyses could not be performed reliably, potentially limiting the detection of activation foci. In addition, the included studies generally reported only statistically significant activation clusters for their specific contrasts, and rarely provided information on deactivations or baseline-referenced effects. Because contrasts differed substantially across studies (e.g., distorted > real, fat > thin, body > house), directionality cannot be meaningfully synthesised across paradigms. As a result, our review focuses on convergent regional involvement rather than up- or down-regulation, which cannot be inferred from the available data.

Additionally, given that five studies were conducted in Eastern populations and four in Western populations, it is possible that some of the observed differences reflect cultural variations in perceptual processing. Indeed, evidence suggests that East Asian individuals generally engage in more holistic face processing, whereas Western individuals focus more on discrete facial features [100]. Furthermore, these differences may also arise from cultural variations in self-construal and attention [101]. In Western cultures, more independent self-construals encourage self-focused attention, supporting stronger self-representations. In contrast, the interdependent self-construals typical of Eastern cultures may lessen self-directed processing, weakening facial self-representations [102]. These cultural differences in perceptual style are likely reflected in neural patterns of self-recognition and may similarly influence how bodies are processed and represented [103–105]. As such, further research is needed to fully understand cultural differences in body representation and the neural mechanisms underlying them. Finally, the included studies predominantly consisted of female participants, limiting our ability to generalize findings across genders. Given that gender differences in body image and related processing have been documented [106,107], future studies should strive for a more balanced representation, as it is unclear if gender differences would be expected with BSE.

Going forward, it will be important for future research to address these limitations by adopting more standardized task paradigms and by incorporating emerging methodological advancements, such as active interaction and adjustment of 3D body stimuli. Comparative studies that directly examine the impact of task type—such as own-body photograph distortions versus virtual avatar comparisons—are also needed. Extending this work to include more gender-balanced non-clinical samples will be important. Once the neurobiological mechanisms of BSE are better understood, these can then be studied in clinical populations, which will be essential for delineating the unique neural mechanisms that contribute to BSE and its role in the development and maintenance of body image disorders.

## 5. Conclusions

In sum, this review synthesized existing fMRI research on BSE in healthy individuals, revealing considerable heterogeneity across studies in terms of tasks, contrasts, and reported regions. Nevertheless, several notable patterns emerged, including frequent involvement of body-selective visual areas (e.g., FBA), dorsal / parietal areas, and prefrontal regions. These findings suggest recurring involvement of several perceptual and higher-order cognitive regions during BSE tasks. However, due to methodological variability, small sample sizes, and limited demographic diversity, current evidence has restricted generalizability and is insufficient to characterize a unified or coherent BSE network. By synthesizing across studies, this review helps clarify where future work is most needed, in particular, the need for standardized, ecologically valid paradigms, combined with larger and more diverse samples. This work will be essential for advancing our understanding of the neural mechanisms underlying BSE and in turn, this knowledge can provide a normative benchmark for interpreting alterations in clinical populations with body image disturbances.

## Supplementary Material

1

## Figures and Tables

**Fig. 1.**
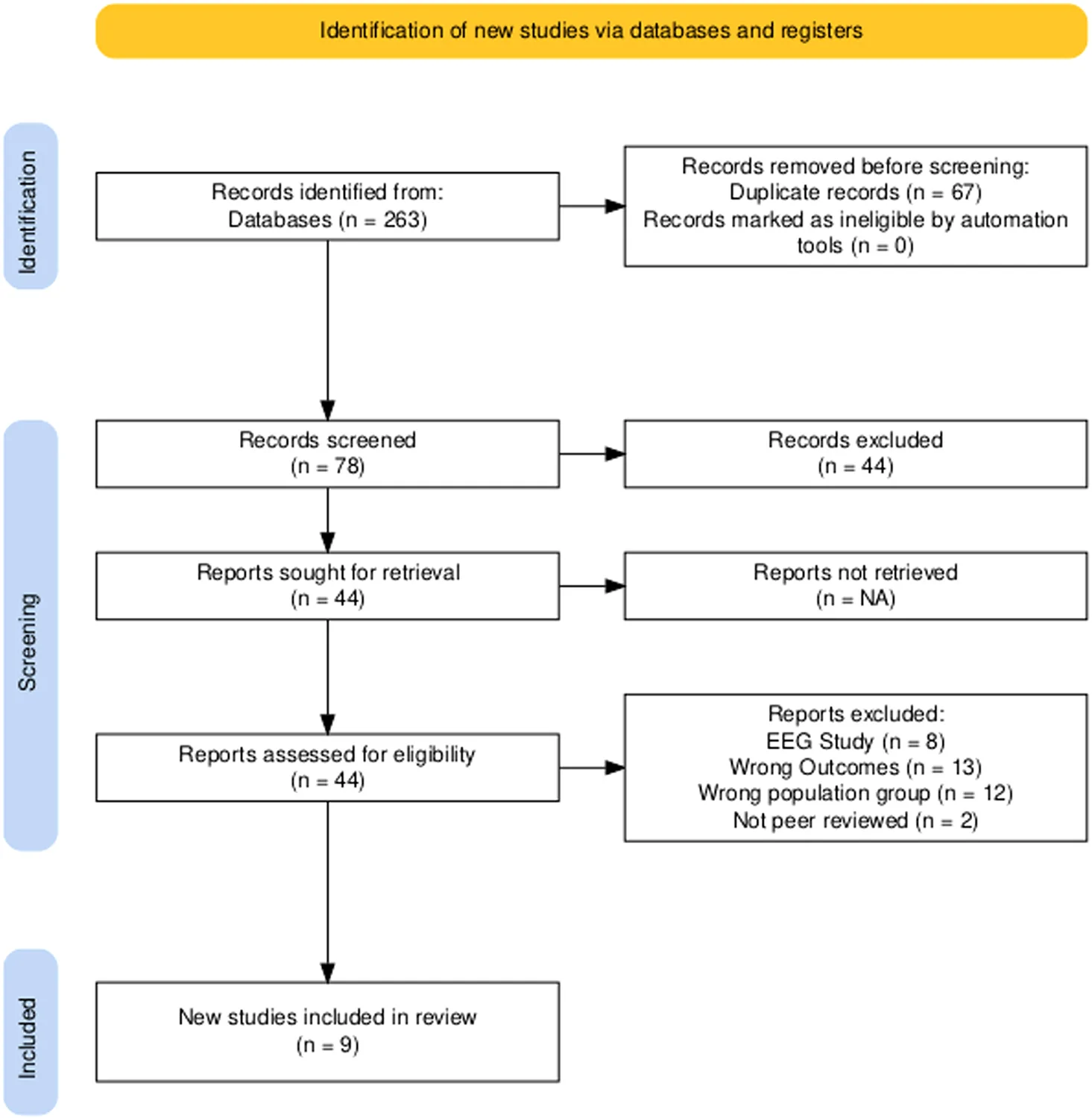
PRISMA flowchart depicting the selection process to reach the final number of included studies in the review.

**Table 1:** Characteristics of nine fMRI studies included in the meta-analysis.

Study	n	Mean age (± SD)	Age range	Gender	Task type	Task and (control) details
Kurosaki et al. (2006) [45]	11	24.50 ± 3.40	NA	F	Distorted own body	Distorted oversized own body image (real own body)
	11	24.80 ± 3.10	NA	M		
Friederich et al. (2007) [46]	18	23.10 ± 6.10	16–35	F	Comparison of own body to others	Other body comparison (interior home object size comparison task)
Miyake et al. (2010) [47]	11	26.50 ± 5.50	NA	F	Distorted own body	Distorted *oversized* own body image (real own body)
						Distorted *undersized* own body image (real own body)
Owens et al. (2010) [48]	10	NA	18–30	F	Comparison of own body to others	Own body avatar comparison (scrambled avatar images)
	9	NA	18–30	M		
Spangler & Allen. (2012) [49]	12	NA	18–30	F	Comparison of own body to others	Own body avatar comparison (scrambled avatar images)
Castellini et al. (2013) [50]	19	26.44 ± 2.18	NA	F	Distorted own body	Distorted own body image (house task)
Hashimoto & Iriki (2013) [51]	11	21.10 ± 2.30	NA	F	Distorted own body	Distorted own body image - horizontal thickness Distorted own body image - dorsoventral thickness
Gao et al. (2016) [52]	13	20.15 ± 1.52	NA	F	Comparison of own body to others	Own body avatar comparison (scrambled body)
Hamamoto et al. (2023) [27]	36	20.90 ± 1.62	NA	F	Distorted own body	Own body comparison with width-distorted silhouette of own body (shape width judgement)

Note. n = sample size; NA = not available; M = male; F = female.

**Table 2:** Principal imaging findings from within-group analyses in each study.

Study	Principal Findings Including Contrast
Kurosaki et al. (2006) [45]	Fat > Thin: Women: BL prefrontal cortex (IFG, MFG), L amygdala, L precentral gyrus; Men: R occipital lobe (middle occipital, lingual gyrus), R superior temporal gyrus, R precuneus. Thin > Fat: Women: L IFG and superior frontal gyrus, L cingulate gyrus and insula; Men: L occipital lobe (inferior occipital, lingual, fusiform gyrus), R fusiform gyrus, R precuneus.
Friederich et al. (2007) [46]	Body > House: BL lateral fusiform gyrus, R inferior parietal lobule, R DLPFC/precentral gyrus, L anterior cingulate.
Miyake et al. (2010) [47]	Fat > Real: R temporal fusiform gyrus, R putamen, R amygdala, BL DLPFC, R IPL, R ventrolateral prefrontal cortex, R premotor cortex, L mPFC, and L parahippocampal gyrus/thalamus. Thin > Real: R DLPFC, BL occipitotemporal (fusiform) gyrus, and R IPL.
Owens et al. (2010) [48]	Thin > Fat: Women: BL MFG Men: R precentral gyrus, R IFG, and medial superior precuneus. Fat > Thin: Women: BL medial superior frontal gyri (mPFC), R frontal operculum; Men: none.
Spangler & Allen. (2012) [49]	Thin > Fat: DLPFC, specifically in the BL middle frontal gyri. Fat > Thin: BL mPFC (outside the cingulate gyrus), including R IFG (near the border of pars triangularis and pars opercularis).
Castellini et al. (2013) [50]	Oversize, undersize, and normal-size conditions: BL fusiform gyrus, inferior temporal gyrus, inferior occipital gyrus, and lingual gyrus, and BL parahippocampal gyrus. Undersize trials: R superior and IPL, L parahippocampal gyrus, and R cuneus.
Hashimoto & Iriki (2013) [51]	Horizontal width distortions: L secondary somatosensory cortex and superior temporal gyrus, and R superior temporal gyrus. Dorsoventral distortions: None.
Gao et al. (2016) [52]	Fat > Control & Thin > Control: Perceptive: BL fusiform gyrus, SPL/IPL, middle/inferior occipital gyrus, precuneus Affective: BL IFG/MFG, DLPFC, parahippocampal gyrus, amygdala mPFC: BL mPFC activated in overweight group for both contrasts; in average-weight group, only for thin > control
Hamamoto et al. (2023) [27]	Actual body comparisons: L anterior cingulate cortex Ideal-body comparisons: R TPJ

*Note. BL* bilateral, *L* left, *R* right, *IFG* inferior frontal gyrus, *MFG* middle frontal gyrus, *DLPFC* dorsolateral prefrontal cortex, *mPFC* medial prefrontal cortex, *TPJ* temporoparietal junction.

## Data Availability

All data is available in supplement.

## Footnotes

2 All neuroimaging modalities such as EEG and TMS were initially included in our registered protocol; however, because their outcome measures are not directly comparable to those of fMRI studies, they could not be synthesised within the current review. This represents a deviation from the original registration. We plan to evaluate EEG findings separately in a future review.

3 We included one study by Friederich et al. (2007) with participants aged 16–35, though the mean age was 23.

## Appendix A. Supporting information

Supplementary data associated with this article can be found in the online version at doi:10.1016/j.bbr.2025.116020.
