# Supplementary material for: Neural correlates of body size estimation: A systematic review and narrative synthesis
Source: Behav Brain Res. Author manuscript; Available in PMC 2026 Oct 13. (PMC13659634; doi:10.1016/j.bbr.2025.116020)
Supplement: Supplementary file 1 [file NIHMS2188753-supplement-1.docx]

**Supplementary Material**

**Title**

Neural correlates of body size estimation: A systematic review and narrative synthesis

**Authors**

Hayden J. Peel^1, 2^*, Akansha M. Naraindas^3^*, Joyce Guo^4^, Valentina Cazzato^5,6^, Jamie D. Feusner^1, 7, 8^

^1^Centre for Addiction and Mental Health, Toronto, ON, Canada

^2^La Trobe University, Melbourne, Victoria, Australia,

^3^Centre for Public Health, Queens University Belfast, United Kingdom

^4^University of California San Diego, San Diego, California

^5^Department of Cognitive Sciences, Psychology, Education, and Cultural Studies, University of Messina, Messina, Italy

^6^Faculty of Health, Liverpool John Moores University, Liverpool, United Kingdom

^7^Department of Psychiatry, Division of Neurosciences & Clinical Translation, University of Toronto, Toronto, ON, Canada

^8^Department of Women’s and Children’s Health, Karolinska Institutet, Stockholm, Sweden

*Denotes co-first authorship

**Supplementary Methods**

1. Search strings for different databases
2. Table of excluded studies at full text and reasons for exclusion
3. Exploratory activation likelihood estimation (ALE) meta-analysis

**Supplementary Results**

**Table S1** Excluded studies title, author(s), published year, DOI, and associated notes for exclusion

**Table S2** Data extraction table

**Table S3** Clusters of significant activity associated with BSE

**Figure S1** Significant clusters of activity from the ALE meta-analysis

**Supplementary Methods**

**1.**	**Search strings for different databases**

*PUBMED* - ("body image" OR "body image distortion" OR "body size estimation" OR "body shape" OR "body thickness") AND ("functional magnetic resonance imaging" OR "functional neuroimaging" OR "functional MRI" OR "fMRI" OR "structural MRI" OR "gray matter" OR "brain volume") AND ("eating disorders" OR "anorexia nervosa" OR "bulimia nervosa" OR "body dysmorphic disorder")

*Web of Science* - TS=("body image" OR "body image distortion" OR "body size estimation" OR "body shape" OR "body thickness")

AND TS=("functional magnetic resonance imaging" OR "functional neuroimaging" OR "functional MRI" OR "fMRI" OR "structural MRI" OR "gray matter" OR "brain volume")

AND TS=("eating disorders" OR "anorexia nervosa" OR "bulimia nervosa" OR "body dysmorphic disorder")

*PsychINFO -* TX("body image" OR "body image distortion" OR "body size estimation" OR "body shape" OR "body thickness")

AND TX("functional magnetic resonance imaging" OR "functional neuroimaging" OR "functional MRI" OR "fMRI" OR "structural MRI" OR "gray matter" OR "brain volume")

AND TX("eating disorders" OR "anorexia nervosa" OR "bulimia nervosa" OR "body dysmorphic disorder")

2. **Table of excluded studies at full text and reasons for exclusion**

**Table S1** Excluded studies title, author(s), published year, DOI, and associated notes for exclusion

| Title | Authors | Year | DOI | Notes |
| --- | --- | --- | --- | --- |
| Neural mechanisms of perceptive and affective processing of body stimuli in anorexia nervosa - are there developmental effects? | Horndasch et al. | 2020 | 10.1016/j.psychres.2020.112853 | Exclusion reason: wrong population group: no within group results; |
| Neural correlates of body comparison and weight estimation in weight-recovered anorexia nervosa: a functional magnetic resonance imaging study | Kodama et al. | 2018 | 10.1186/s13030-018-0134-z | Exclusion reason: wrong population group:no within group results; |
| Body image distortions in bulimia nervosa: investigating body size overestimation and body size satisfaction by fMRI | Mohr et al. | 2011 | 10.1016/j.neuroimage.2011.02.069 | Exclusion reason: wrong population group: |
| Separating two components of body image in anorexia nervosa using fMRI | Mohr et al. | 2010 | 10.1017/s0033291709991826 | Exclusion reason: wrong population group: |
| Visual body size estimation in adolescent anorexia nervosa: Behavioural and neurophysiological data suggest intact visual perception and biased emotional attention | Rahder et al. | 2023 | 10.21203/rs.3.rs-2999413/v1 | Exclusion reason: study is not peer reviewed; |
| Changes in neuronal correlates of body image processing by means of cognitive-behavioural body image therapy for eating disorders: a randomized controlled fMRI study | Vocks et al. | 2011 | 10.1017/S0033291710002382 | Exclusion reason: Wrong outcomes;treatment before and after study; |
| Neural correlates of human body perception | Aleong, & Paus | 2010 | 10.1162/jocn.2009.21211 | Exclusion reason: Wrong outcomes; BODY SIZE ADAPTATION |
| Neural correlates of viewing photographs of one's own body and another woman's body in anorexia and bulimia nervosa: an fMRI study | Vocks et al. | 2010 | 10.1503/jpn.090048 | Exclusion reason: Wrong outcomes; a self-recognition task more than a BSE task; |
| Distinct neural response to visual perspective and body size in the extrastriate body area | Carey et al. | 2019 | 10.1016/j.bbr.2019.112063 | Exclusion reason: Wrong outcomes; |
| Investigation of Brain Activation Patterns Related to the Feminization or Masculinization of Body and Face Images across Genders | Ceruti et al. | 2022 | 10.3390/tomography8040176 | Exclusion reason: Wrong outcomes; not a BSE task |
| Body image distortion reveals amygdala activation in patients with anorexia nervosa â€“ a functional magnetic resonance imaging study | Seeger et al. | 2002 | https://doi.org/10.1016/S0304-3940(02)00312-9 | Exclusion reason: wrong population group: |
| Brain activation and connectivity in anorexia nervosa and body dysmorphic disorder when viewing bodies: relationships to clinical symptoms and perception of appearance | Moody et al. | 2021 | 10.1007/s11682-020-00323-5 | Exclusion reason: wrong population group: |
| Differential neuronal responses to the self and others in the extrastriate body area and the fusiform body area | Vocks et al. | 2010 | 10.3758/cabn.10.3.422 | Exclusion reason: Wrong outcomes; self vs other discrimination task, unsure if BSE takes place here; |
| Distinct cortical networks for the detection and identification of human body | Hodzic et al. | 2009 | https://doi.org/10.1016/j.neuroimage.2009.01.027 | Exclusion reason: Wrong outcomes; self-recognition effect as opposed to BSE effect; |
| Effects of body image therapy on the activation of the extrastriate body area in anorexia nervosa: an fMRI study | Vocks et al. | 2010 | 10.1016/j.pscychresns.2010.05.011 | Exclusion reason: Wrong outcomes; |
| Functional neuroanatomy of body checking in people with anorexia nervosa | Suda et al. | 2013 | 10.1002/eat.22150 | Exclusion reason:wrong population group: |
| Functional neuroanatomy of body shape perception in healthy and eating-disordered women | Uher et al. | 2005 | 10.1016/j.biopsych.2005.06.001 | Exclusion reason: Wrong outcomes; Ps showed bodies of different shapes with no explicit instruction to compare or make body based size judgements; |
| Gender differences in brain activity generated by unpleasant word stimuli concerning body image: an fMRI study | Shirao et al. | 2005 | 10.1192/bjp.186.1.48 | Exclusion reason: Wrong outcomes; body words not BSE |
| A Neural Signature of Anorexia Nervosa in the Ventral Striatal Reward System | Fladung et al. | 2010 | 10.1176/appi.ajp.2009.09010071 | Exclusion reason: wrong population group: no within group results; |
| Temporomesial activation in young females associated with unpleasant words concerning body image | Shirao et al. | 2003 | 10.1159/000073630 | Exclusion reason: Wrong outcomes; body words not BSE |
| Wagner A, Ruf M, Braus DF, Schmidt MH. Neuronal activity changes and body image distortion in anorexia nervosa. NeuroReport 14: 2193-2197 | Wagner et al. | 2004 | 10.1097/01.wnr.0000089567.45990.d9 | Exclusion reason: study is not peer reviewed |
| The undue influence of shape and weight on self-evaluation in anorexia nervosa, bulimia nervosa and restrained eaters: a combined ERP and behavioral study | Blechert et al. | 2011 | 10.1017/S0033291710000395 | Exclusion reason: EEG study |
| Self and other body perception in anorexia nervosa: The role of posterior DMN nodes | Via et al. | 2018 | 10.1080/15622975.2016.1249951 | Exclusion reason: Wrong outcomes;task not BSE, Involves looking at own body and other body; |
| Neural processing of negative word stimuli concerning body image in patients with eating disorders: An fMRI study | Miyake et al. | 2010 | 10.1016/j.neuroimage.2009.12.095 | Exclusion reason: Wrong outcomes: attractiveness judgements not BSE judgements |
| Neural Correlates and Perceived Attractiveness of Male and Female Shoulder-to-Hip Ratio in Men and Women: An EEG Study | Pazhoohi et al. | 2023 | 10.1007/s10508-023-02610-w | Exclusion reason: EEG study |
| Sustained hypervigilance for one’s own body in women with weight and shape concerns: Competition effects in early visual processing investigated by steady-state visual evoked potentials (SSVEP) | Voges et al. | 2019 |  | Exclusion reason: EEG study |
| Neural correlates of appearance-based social comparison: The modulating effects of body dissatisfaction and person perspective | Wang et al. | 2019 | 10.1016/j.biopsycho.2019.03.007 | Exclusion reason: EEG study |
| Somatosensory cortical representation of the body size | Giurgola et al. | 2019 | 10.1002/hbm.24614 | Exclusion reason: EEG study |
| Neural correlates of distorted body images in adolescent girls with anorexia nervosa: How is it different from major depressive disorder? | Karaku et al. | 2024 | 10.1111/jnp.12340 | Exclusion reason: wrong population group Adolescent sample |
| Neuronal activity changes and body image distortion in anorexia nervosa | Wagner et al. | 2003 | 10.1097/00001756-200312020-00012 | Exclusion reason: wrong population group Adolescent sample |
| Neural correlates of body dissatisfaction in anorexia nervosa | Friederich et al | 2010 | 10.1016/j.neuropsychologia.2010.04.036 | Exclusion reason: wrong population group: |
| Visualizing Emotional Arousal within the Context of Body Size Evaluation: A Pilot Study of Steady-State Visual Evoked Potentials in Women with Anorexia Nervosa and Healthy Controls | Henn et al. | 2022 | 10.1016/j.biopsycho.2019.02.010 | Exclusion reason: EEG study |
| Appearance concerns are uniquely associated with LPP amplitude to pictures of oneself | Jordan et al. | 2022 | 10.1093/scan/nsab115 | Exclusion reason: EEG study |
| Attention biases in preoccupation with body image: An ERP study of the role of social comparison and automaticity when processing body size | Uusberg et al. | 2018 | https://doi.org/10.1016/j.biopsycho.2018.03.007 | Exclusion reason: EEG study |
| Reduced body-image disturbance by body-image interventions is associated with neural-response changes in visual and social processing regions: a preliminary study | Hamamoto et al. | 2024 | https://doi.org/10.3389/fpsyt.2024.1337776 | Exclusion reason: wrong population group: no within group results; |

**2.**	**Exploratory activation likelihood estimation (ALE) meta-analysis**

A limitation of the ALE analysis in this review is its low statistical power, arising from the limited number of studies and sample sizes (1). Although the ALE approach is effective at identifying statistically significant convergence across studies, insufficient statistical power limits its ability to detect meaningful activations. We present the results here for the sake of completeness and in alignment with our PROSPERO-registered protocol.

*Data analysis:* ALE tests whether spatial convergence of activation foci reported in studies is greater than that expected by random chance (2-4). ALE models reported foci peaks in studies as peaks of three-dimensional Gaussian distributions to account for spatial uncertainty inherent in the data, which is influenced by factors such as sample size (e.g., smaller studies produce broader Gaussians) (3). For each study, a modeled activation map is created by merging the probability distributions for all foci, controlling effects of multiple foci being reported close together (5). ALE scores are then calculated at each voxel by taking the union of these modeled activation maps, generating a map of convergence (2). Statistical significance of this convergence is tested by comparing ALE scores against a null distribution generated through random resampling of the foci under the assumption of spatial independence. All coordinates were converted into Montreal Neurological Institute (MNI) space using the automatic conversion routines in GingerALE with the icbm2tal transform (brainmap.org). Cluster-level family-wise error (FWE) correction was applied to control for multiple comparisons, using an uncorrected voxel-wise threshold of *p* < .001 and a cluster-wise threshold of *p* < .05 (6).

*Results:* The ALE results from the nine studies associated with BSE with three clusters. This included one cluster in the right FBA, one in the right inferior frontal junction (IFJ), and one which included the right inferior frontal gyrus (IFG) and middle frontal gyrus (MFG) (see Table S2, and Figure S1).

While three clusters survived correction, these results should be interpreted with caution due to the limited statistical power inherent in ALE analyses with fewer than 17 experiments (1). We include the findings here to maintain transparency with our PROSPERO-registered protocol, but refrain from drawing strong inferences based on this analysis.

| **Table S2** Data extraction table | | | | | | | | | | |
| --- | --- | --- | --- | --- | --- | --- | --- | --- | --- | --- |
| **Name (year**) | **N (sex) age (*M*** ± **SD/range)** | **Country** | **Manipulation** | **MNI coordinates**  **x y z** | | | ***p* value** | ***t*** | **Software** | **Contrasts** |
| Hamamoto et al (2023) | 36 (F) 20.9 ± 1.62) | Japan | own body compare | -14 | 42 | 16 | .004 | 4.82 | SPM | actual-body task> control task |
| (a) Owens et al (2010) | 10 (F) 18-30 | USA | own body compare | 45 | 21 | 33 | < .001 | 4.48 | SPM | thin > fat |
|  |  |  |  | -50 | 30 | 33 |  | 4.47 |  |  |
|  |  |  |  | -10 | 56 | 29 |  | 4.92 |  | fat > thin |
|  |  |  |  | 13 | 47 | 24 |  | 4.82 |  |  |
|  |  |  |  | 53 | 13 | 4 |  | 4.45 |  |  |
|  |  |  |  | 53 | 23 | 9 |  | 4.49 |  |  |
| (b) Owens et al (2010) | 9 (M) 18-30 | USA | own body compare | 52 | -2 | 45 | < .001 | 5.77 | SPM | thin>fat |
|  |  |  |  | 52 | 35 | 10 |  | 5.77 |  |  |
|  |  |  |  | 0 | 68 | 60 |  | 6.87 |  |  |
|  |  |  |  | - | - | - |  | - |  | fat>thin |
| Miyake et al (2010) | 11 (F) 26.5 ± 5.5 | Japan | distorted oversized/undersized own body image | 46 | − 44 | − 20 | < .001 | 8.98 | SPM | fat> real |
|  |  |  |  | 30 | − 4 | − 8 |  | 6.92 |  |  |
|  |  |  |  | 34 | 0 | − 12 |  | 6.25 |  |  |
|  |  |  |  | − 10 | 40 | 34 |  | 8.98 |  |  |
|  |  |  |  | − 32 | 22 | 40 |  | 7.67 |  |  |
|  |  |  |  | 48 | − 52 | 34 |  | 9.47 |  |  |
|  |  |  |  | − 16 | − 32 | 8 |  | 7.28 |  |  |
|  |  |  |  | 52 | 24 | − 18 |  | 5.36 |  |  |
|  |  |  |  | 44 | − 16 | 50 |  | 8.98 |  |  |
|  |  |  |  | 52 | 12 | 30 |  | 5.36 |  | thin>real |
|  |  |  |  | 44 | − 56 | − 22 |  | 9.47 |  |  |
|  |  |  |  | − 40 | − 48 | − 14 |  | 6.24 |  |  |
|  |  |  |  | 34 | − 54 | 40 |  | 9.00 |  |  |
| Hashimoto et al (2013) | 11 (F) 21.1 ± 2.3 | Japan | horizontal width distortion/dorsoventral thickness | ^-55 | -12 | 2 |  | 13.86 | SPM |  |
|  |  |  |  | ^46 | -32 | 11 |  | 12.86 |  |  |
|  |  |  |  | -12 | -24 | 66 |  | 6.21 |  |  |
|  |  |  |  | -42 | -44 | 8 |  | 6.21 |  |  |
|  |  |  |  | 38 | -20 | 25 |  | 6.18 |  |  |
|  |  |  |  | -18 | -58 | 12 |  | 6.12 |  |  |
|  |  |  |  | 10 | 20 | 56 |  | 5.73 |  |  |
|  |  |  |  | 48 | -54 | 16 |  | 5.70 |  |  |
|  |  |  |  | 32 | -19 | 47 |  | 5.44 |  |  |
|  |  |  |  | 51 | -1 | -13 |  | 5.07 |  |  |
|  |  |  |  | 20 | 6 | 51 |  | 4.90 |  |  |
|  |  |  |  | -32 | -28 | -22 |  | 6.21 |  |  |
|  |  |  |  | 10 | -70 | 7 |  | 6.05 |  |  |
|  |  |  |  | 48 | -39 | 42 |  | 4.59 |  |  |
| Spangler and Allen (2012) | 12 (F) 18-30 | USA | own body compare | 45 | 20 | 38 | < .001 | 4.48 | SPM | thin>fat |
|  |  |  |  | -47 | 30 | 37 |  | 4.47 |  |  |
|  |  |  |  | -10 | 56 | 29 |  | 4.92 |  | fat>thin |
|  |  |  |  | 13 | 50 | 24 |  | 4.82 |  |  |
|  |  |  |  | 53 | 23 | 9 |  | 4.49 |  |  |
| (a) Gao et al. (2016) (normal weight group) | 13 (F) 20.15 ± 1.62 | China | own body compare | -1.38 | 13.1 | 62.5 | < .05 | 9.27 | Brainvoyager | fat>control |
|  |  |  |  | 43.23 | 33.8 | 5.9 |  | 5.72 |  |  |
|  |  |  |  | -33.97 | 5.05 | 47.04 |  | 8.89 |  |  |
|  |  |  |  | 37.37 | -4.27 | 46.75 |  | 7.54 |  |  |
|  |  |  |  | -34.33 | 15.52 | 21.36 |  | 5.64 |  |  |
|  |  |  |  | 53.29 | 13.37 | 27.92 |  | 10.77 |  |  |
|  |  |  |  | 4.01 | 31.17 | -20.06 |  | 4.2 |  |  |
|  |  |  |  | -15.3 | -4 | -16.26 |  | 5.79 |  |  |
|  |  |  |  | -17.25 | 14.03 | 4.4 |  | 5.95 |  |  |
|  |  |  |  | 36.36 | -5.27 | -32.7 |  | 5.78 |  |  |
|  |  |  |  | -18.37 | -5.95 | -2.56 |  | 6.15 |  |  |
|  |  |  |  | 17.16 | -6.76 | -13.17 |  | 5.79 |  |  |
|  |  |  |  | -4.92 | -51.59 | 25.25 |  | 4.02 |  |  |
|  |  |  |  | -18.24 | -27.98 | 2.97 |  | 6.14 |  |  |
|  |  |  |  | 17.37 | -28.16 | -0.97 |  | 4.71 |  |  |
|  |  |  |  | -60.67 | -7.35 | -15.15 |  | 5.54 |  |  |
|  |  |  |  | -20.74 | -65.1 | 53.76 |  | 13.94 |  |  |
|  |  |  |  | -33.72 | -55.47 | 54.14 |  | 8.32 |  |  |
|  |  |  |  | -24.15 | -75.18 | 38 |  | 9.42 |  |  |
|  |  |  |  | 18.15 | -58.48 | 53.56 |  | 9.22 |  |  |
|  |  |  |  | -42.24 | -49.49 | -19.14 |  | 8.22 |  |  |
|  |  |  |  | 36.58 | -35.38 | -21.88 |  | 10.67 |  |  |
|  |  |  |  | -44.86 | -47.06 | 29.97 |  | 5.01 |  |  |
|  |  |  |  | -46.09 | -64.57 | 14.91 |  | 11.59 |  |  |
|  |  |  |  | 52.75 | 69.48 | -3.39 |  | 12.65 |  |  |
|  |  |  |  | -40.75 | -65.07 | 9.27 |  | 11.16 |  |  |
|  |  |  |  | 43.49 | -64.98 | 5.59 |  | 11.52 |  |  |
|  |  |  |  | -40.84 | -76.64 | 0.33 |  | 18.21 |  |  |
|  |  |  |  | 43.4 | -76.55 | -3.35 |  | 27.71 |  |  |
|  |  |  |  | 30.09 | -75.9 | -30.09 |  | 5.64 |  |  |
|  |  |  |  | 1.68 | 16.12 | 48.7 |  | 7.92 |  | thin > control |
|  |  |  |  | 6.28 | 10.56 | 69.34 |  | 7.29 |  |  |
|  |  |  |  | 0.84 | 67.81 | -6.83 |  | 6.29 |  |  |
|  |  |  |  | 5.07 | 57.32 | -15.95 |  | 7.61 |  |  |
|  |  |  |  | -52.56 | 10.97 | 31.08 |  | 8.02 |  |  |
|  |  |  |  | 58.72 | 11.36 | 29.15 |  | 7.33 |  |  |
|  |  |  |  | 44.4 | 34.43 | 12.54 |  | 8.24 |  |  |
|  |  |  |  | -11.91 | -48.42 | 71 |  | 7.43 |  |  |
|  |  |  |  | 0.89 | 10.2 | -14.57 |  | 5.2 |  |  |
|  |  |  |  | -0.6 | -46.04 | 26.87 |  | 4.02 |  |  |
|  |  |  |  | -0.65 | 26.88 | 37.59 |  | 7.13 |  |  |
|  |  |  |  | 7.85 | 12.69 | 23.16 |  | 5.44 |  |  |
|  |  |  |  | 6.26 | -71.7 | 50.59 |  | 7.25 |  |  |
|  |  |  |  | -17.13 | 10.5 | 12.6 |  | 5.29 |  |  |
|  |  |  |  | 19.62 | 5.31 | 12.48 |  | 4.8 |  |  |
|  |  |  |  | -9.72 | -0.4 | -1.02 |  | 6.29 |  |  |
|  |  |  |  | -32.79 | -3.38 | -31.71 |  | 5.74 |  |  |
|  |  |  |  | 30.96 | -3.06 | -31.7 |  | 6.65 |  |  |
|  |  |  |  | -10.62 | -15.52 | 9.46 |  | 5.61 |  |  |
|  |  |  |  | 15.29 | -15.53 | 7.9 |  | 5.73 |  |  |
|  |  |  |  | -0.96 | 60.62 | 19.69 |  | 5.19 |  |  |
|  |  |  |  | 6.38 | -63.11 | 62.06 |  | 6.48 |  |  |
|  |  |  |  | 1.54 | -59.32 | 22.55 |  | 8.57 |  |  |
|  |  |  |  | -15.79 | -84.88 | 14.17 |  | 9.05 |  |  |
|  |  |  |  | -21.22 | -88.4 | 11.25 |  | 15.18 |  |  |
|  |  |  |  | -2.03 | 60.72 | 20.82 |  | 4.3 |  | fat>thin |
|  |  |  |  | -14.82 | -95.59 | 2.88 |  | 7.09 |  |  |
|  |  |  |  | 14.39 | -95.18 | 5.71 |  | 7.25 |  |  |
|  |  |  |  | 27.01 | -84.55 | -19.09 |  | 4.79 |  |  |
|  |  |  |  | 3.84 | 16.13 | 48.66 |  | 4.84 |  | thin>fat |
|  |  |  |  | -22.11 | 4.88 | 44.61 |  | 5.69 |  |  |
|  |  |  |  | -18.74 | 31.74 | -24.22 |  | 4.79 |  |  |
|  |  |  |  | 1.54 | 31.45 | 40.46 |  | 5.21 |  |  |
|  |  |  |  | 3.71 | 29.33 | 40.63 |  | 4.74 |  |  |
|  |  |  |  | -24.9 | 33.04 | 1.53 |  | 5.03 |  |  |
|  |  |  |  | 33.43 | 27.72 | -1.17 |  | 4.96 |  |  |
|  |  |  |  | 29.16 | 14.94 | 0.16 |  | 5.92 |  |  |
|  |  |  |  | -33.84 | -10.83 | -30.95 |  | 6.14 |  |  |
|  |  |  |  | -35.96 | -8.3 | -26.68 |  | 5.65 |  |  |
|  |  |  |  | 33.11 | -3.15 | -32.85 |  | 5.13 |  |  |
|  |  |  |  | 1.43 | 14.25 | 28.72 |  | 3.94 |  |  |
|  |  |  |  | 3.65 | 7.02 | 31.64 |  | 5.41 |  |  |
|  |  |  |  | -3.51 | 39.11 | -15.12 |  | 5.06 |  |  |
|  |  |  |  | 19.13 | 42.17 | -18.05 |  | 5.45 |  |  |
|  |  |  |  | 1.41 | -62.49 | 11.66 |  | 5 |  |  |
|  |  |  |  | 8.87 | -12.91 | 13.35 |  | 5.48 |  |  |
|  |  |  |  | -31.6 | -10.2 | -24.33 |  | 4.05 |  |  |
|  |  |  |  | -31.38 | -33.73 | -11.92 |  | 6.22 |  |  |
|  |  |  |  | -18.17 | -40.63 | 5.35 |  | 5.58 |  |  |
|  |  |  |  | -23.19 | 6.66 | -39.59 |  | 4.44 |  |  |
|  |  |  |  | -31.37 | -43.51 | -13.19 |  | 7.69 |  |  |
|  |  |  |  | 36.6 | -35.18 | -19.66 |  | 8.21 |  |  |
|  |  |  |  | -16.25 | -51.83 | 69.17 |  | 5.72 |  |  |
|  |  |  |  | 11.8 | -60.76 | 63.98 |  | 5 |  |  |
|  |  |  |  | -30.67 | -37.15 | 43.31 |  | 6.7 |  |  |
|  |  |  |  | -8.9 | -56.66 | 51.6 |  | 7.05 |  |  |
|  |  |  |  | 1.41 | -76.73 | 8.58 |  | 4.12 |  |  |
|  |  |  |  | 3.61 | -83 | 10.29 |  | 4.09 |  |  |
|  |  |  |  | -30.84 | -88.77 | 19.29 |  | 5.19 |  |  |
|  |  |  |  | -31.14 | -91.06 | -5.13 |  | 6.49 |  |  |
|  |  |  |  | -9.84 | -63.72 | -23.89 |  | 5.52 |  |  |
|  |  |  |  | 0.84 | -47.08 | -30.2 |  | 5 |  |  |
| (b) Gao et al. (2016) (overweight group) | 13 (F) 20.31 ± 1.70 | China | own body compare | -46.59 | 48.71 | -1.89 |  | 4.83 | Brainvoyager | fat>control |
|  |  |  |  | 51.85 | 44.57 | 5.81 |  | 8.99 |  |  |
|  |  |  |  | -49.6 | 28.57 | 12.48 |  | 5.85 |  |  |
|  |  |  |  | 45.44 | 25.4 | 7.81 |  | 7.1 |  |  |
|  |  |  |  | -31.12 | -24.1 | 10.66 |  | 6.27 |  |  |
|  |  |  |  | 38.65 | 32.88 | -15.22 |  | 5.05 |  |  |
|  |  |  |  | -50.35 | 12.46 | 35.38 |  | 7.01 |  |  |
|  |  |  |  | 54.39 | 9.12 | 28.32 |  | 11.13 |  |  |
|  |  |  |  | -3.69 | 13.01 | 50.22 |  | 8.14 |  |  |
|  |  |  |  | 8.34 | 6.54 | 60.74 |  | 5.92 |  |  |
|  |  |  |  | -37.4 | 16.76 | 34.73 |  | 8.77 |  |  |
|  |  |  |  | 42.52 | 5.89 | 28.85 |  | 6.52 |  |  |
|  |  |  |  | -37.95 | -32.25 | -18.68 |  | 7.93 |  |  |
|  |  |  |  | 36.54 | -32.4 | -24.41 |  | 8.67 |  |  |
|  |  |  |  | -32.74 | -5.2 | -28.17 |  | 5.15 |  |  |
|  |  |  |  | 33.15 | -2.84 | -29.52 |  | 6.51 |  |  |
|  |  |  |  | -14.96 | 7.32 | 12.87 |  | 5.23 |  |  |
|  |  |  |  | -20.41 | -1.83 | 7.15 |  | 6.34 |  |  |
|  |  |  |  | -19.34 | -0.77 | 7.02 |  | 6.28 |  |  |
|  |  |  |  | 20.62 | -0.73 | 5.22 |  | 5.45 |  |  |
|  |  |  |  | -18.26 | -30.31 | 0.97 |  | 5.15 |  |  |
|  |  |  |  | 19.56 | -27.95 | 1.21 |  | 7.67 |  |  |
|  |  |  |  | -32.61 | -53.03 | 57.25 |  | 7.01 |  |  |
|  |  |  |  | 35.41 | -53.1 | 52.73 |  | 6.15 |  |  |
|  |  |  |  | -9.93 | -70.49 | 52.98 |  | 6.47 |  |  |
|  |  |  |  | 8.44 | -72.65 | 51.76 |  | 9.33 |  |  |
|  |  |  |  | -33.71 | -54.31 | 55.15 |  | 8.04 |  |  |
|  |  |  |  | 37.55 | -53.3 | 50.47 |  | 8.13 |  |  |
|  |  |  |  | -21.18 | -91.39 | 13.78 |  | 12.79 |  |  |
|  |  |  |  | 24.2 | -91.23 | 12.99 |  | 10.67 |  |  |
|  |  |  |  | -42.99 | -57.95 | 5.24 |  | 9.24 |  |  |
|  |  |  |  | 45.65 | -57.32 | 7.03 |  | 10.35 |  |  |
|  |  |  |  | -42.23 | -48.32 | -18.14 |  | 8.42 |  |  |
|  |  |  |  | 38.87 | -54.1 | -15.58 |  | 9.64 |  |  |
|  |  |  |  | -34.14 | -91.43 | 14.01 |  | 13.03 |  |  |
|  |  |  |  | 37.22 | -87.47 | 18 |  | 19.28 |  |  |
|  |  |  |  | -33.24 | -92.78 | -0.44 |  | 13.67 |  |  |
|  |  |  |  | 33.7 | -92.85 | -4.94 |  | 11.42 |  |  |
|  |  |  |  | -5.51 | -71.25 | -24.34 |  | 6.91 |  |  |
|  |  |  |  | 5.3 | -75.57 | -25.21 |  | 5.22 |  |  |
|  |  |  |  | -10.64 | 62.07 | 24.19 |  | 4.36 |  | thin>control |
|  |  |  |  | -50.24 | 6.7 | 42.67 |  | 8.37 |  |  |
|  |  |  |  | 51.2 | 6.23 | 32.03 |  | 10.31 |  |  |
|  |  |  |  | -38.76 | 34.34 | 16.21 |  | 4.72 |  |  |
|  |  |  |  | 48.72 | 34.44 | 12.47 |  | 6.14 |  |  |
|  |  |  |  | -52.86 | 22.93 | 9.73 |  | 4.59 |  |  |
|  |  |  |  | 45.43 | 27.53 | 7.6 |  | 4.31 |  |  |
|  |  |  |  | -42.36 | 42.58 | -10.32 |  | 6.13 |  |  |
|  |  |  |  | 37.61 | 35.41 | -10.97 |  | 4.3 |  |  |
|  |  |  |  | -50.44 | 12.9 | 28.61 |  | 6.21 |  |  |
|  |  |  |  | 45.62 | 26.75 | 22.25 |  | 10.47 |  |  |
|  |  |  |  | -4.72 | 17.78 | 55.37 |  | 5.6 |  |  |
|  |  |  |  | 6.16 | 6.33 | 58.56 |  | 4.84 |  |  |
|  |  |  |  | -37.3 | 3.24 | 39.43 |  | 8.78 |  |  |
|  |  |  |  | 51.29 | -1.87 | 37.31 |  | 8.75 |  |  |
|  |  |  |  | -35.98 | -2.98 | -27.21 |  | 6.02 |  |  |
|  |  |  |  | 33.18 | -2.63 | -27.3 |  | 5.11 |  |  |
|  |  |  |  | -17.13 | -26.7 | 5.07 |  | 8.25 |  |  |
|  |  |  |  | -32.61 | -53.03 | 57.25 |  | 6.98 |  |  |
|  |  |  |  | 27.96 | -52.3 | 61.74 |  | 7.624 |  |  |
|  |  |  |  | -24.37 | -56.01 | 24.9 |  | 7.02 |  |  |
|  |  |  |  | 17.14 | -57.97 | 59.13 |  | 8.23 |  |  |
|  |  |  |  | -33.66 | -52.83 | 59.48 |  | 7.34 |  |  |
|  |  |  |  | 34.36 | -52.9 | 54.97 |  | 8.34 |  |  |
|  |  |  |  | -20.07 | -91.17 | 15.98 |  | 10.38 |  |  |
|  |  |  |  | 22.03 | -90.17 | 12.93 |  | 11.44 |  |  |
|  |  |  |  | -43.89 | -63.19 | 18.1 |  | 8.21 |  |  |
|  |  |  |  | 51.23 | -62.54 | 19.78 |  | 9.34 |  |  |
|  |  |  |  | -42.19 | -42.59 | -14.22 |  | 8.42 |  |  |
|  |  |  |  | 42.06 | -39.1 | -16 |  | 8.5 |  |  |
|  |  |  |  | -33.19 | -91.3 | 3.89 |  | 12.39 |  |  |
|  |  |  |  | 34.9 | -84.26 | 6.52 |  | 15.54 |  |  |
|  |  |  |  | -33.24 | -92.78 | -0.44 |  | 11.24 |  |  |
|  |  |  |  | 33.74 | -91.48 | -1.71 |  | 14.09 |  |  |
|  |  |  |  | -7.71 | -74.87 | -28.42 |  | 6.07 |  |  |
|  |  |  |  | 51.98 | 40.08 | 15.22 |  | 5.56 |  | fat>thin |
|  |  |  |  | 19.16 | 10.6 | -22.77 |  | 5.44 |  |  |
|  |  |  |  | 41.48 | 1.84 | 31.5 |  | 6.04 |  |  |
|  |  |  |  | -26.12 | -61.72 | 55.75 |  | 5.91 |  |  |
|  |  |  |  | 34.38 | -61.51 | 54.7 |  | 4.97 |  |  |
|  |  |  |  | 36.66 | 19.98 | -3.83 |  | 4.21 |  |  |
|  |  |  |  | -21.95 | -63.92 | 43.57 |  | 5.85 |  |  |
|  |  |  |  | 28.74 | -60 | 36.72 |  | 5.8 |  |  |
|  |  |  |  | -34.07 | -81.03 | 21.94 |  | 4.98 |  |  |
|  |  |  |  | 31.77 | -81.31 | 15.25 |  | 5.44 |  |  |
|  |  |  |  | -16.09 | -88.23 | -10.15 |  | 7.63 |  |  |
|  |  |  |  | 16.43 | -87.28 | -1.83 |  | 6.41 |  |  |
|  |  |  |  | -39.05 | -66.26 | -26.5 |  | 6.14 |  |  |
|  |  |  |  | -4.51 | 25.49 | -11.51 |  | 5.68 |  | thin>fat |
|  |  |  |  | 0.96 | 21.67 | -6.75 |  | 5.51 |  |  |
|  |  |  |  | 11.3 | -79.8 | 21.05 |  | 5.01 |  |  |
|  |  |  |  | -2.78 | -80.17 | 17.96 |  | 4.21 |  |  |
|  |  |  |  | 58.73 | -50.92 | 17.38 |  | 4.31 |  |  |
| Kurosaki et al (2006) | 11 (F) 24.5 ± 3.4 | Japan | distorted oversized own body image | *35 | -87 | -1 | < .05 | 8.25 |  | fat>real |
|  |  |  |  | *3 | -92 | -11 |  | 6.40 |  |  |
|  |  |  |  | *11 | -83 | -19 |  | 6.28 |  |  |
|  |  |  |  | **-21 | -75 | -31 |  | 8.17 |  |  |
|  |  |  |  | **-30 | -68 | -34 |  | 6.41 |  |  |
|  |  |  |  | **31 | -55 | 32 |  | 6.07 |  |  |
|  |  |  |  | **25 | -71 | 32 |  | 4.61 |  |  |
|  |  |  |  | **17 | -71 | 40 |  | 4.52 |  |  |
|  |  |  |  | *-38 | -82 | -19 |  | 10.93 |  | thin>real |
|  |  |  |  | *-14 | -95 | -26 |  | 10.93 |  |  |
|  |  |  |  | *-29 | -84 | -25 |  | 7.20 |  |  |
|  |  |  |  | *3 | -97 | 4 |  | 10.82 |  |  |
|  |  |  |  | *33 | -69 | -21 |  | 9.06 |  |  |
|  |  |  |  | *37 | -49 | -23 |  | 8.73 |  |  |
|  |  |  |  | *25 | -66 | 31 |  | 5.83 |  |  |
| Castellini et al. (2013) | 19 (F) 26.44  ± 2.18, | Italy | distorted oversized/undersized/actual own body image | 26 | -53 | -13 | < .05 | 7.94 |  |  |
|  |  |  |  | 45 | -68 | -12 |  | 7.22 | SPM | oversize |
|  |  |  |  | 46 | -60 | -9 |  | 7.19 |  |  |
|  |  |  |  | 45 | -48 | -22 |  | 6.84 |  |  |
|  |  |  |  | -45 | -72 | -10 |  | 6.55 |  |  |
|  |  |  |  | -26 | -47 | -12 |  | 6.28 |  |  |
|  |  |  |  | 18 | -99 | 7 |  | 7.42 |  |  |
|  |  |  |  | 46 | -62 | -9 |  | 7.09 |  | undersize |
|  |  |  |  | 39 | -81 | -1 |  | 6.02 |  |  |
|  |  |  |  | 24 | -53 | -13 |  | 6.96 |  |  |
|  |  |  |  | 45 | -44 | 57 |  | 6.64 |  |  |
|  |  |  |  | 27 | -53 | 47 |  | 6.31 |  |  |
|  |  |  |  | -13 | -87 | -13 |  | 9.41 |  |  |
|  |  |  |  | -23 | -56 | -8 |  | 7.23 |  |  |
|  |  |  |  | -41 | -66 | -9 |  | 6.89 |  |  |
|  |  |  |  | -43 | -75 | -10 |  | 6.19 |  |  |
|  |  |  |  | 24 | -55 | -14 |  | 9.18 |  |  |
|  |  |  |  | 20 | 34 | -81 |  | 7.51 |  | normal size |
|  |  |  |  | 20 | 54 | -68 |  | 7.88 |  |  |
|  |  |  |  | 38 | 58 | -76 |  | 7.37 |  |  |
|  |  |  |  | 38 | 58 | -76 |  | 8.49 |  |  |
|  |  |  |  | 38 | -36 | -77 |  | 7.14 |  |  |
| Friederich et al. (2007) | 18 (F) 16-35 | UK | compare their own body/home | -7 | 36 | 3 | .005 | 3.22 |  | XBAM |
|  |  |  |  | 48 | 3 | 33 | .005 | 3.22 |  |  |
|  |  |  |  | 51 | -33 | 42 | .007 | 3.06 |  |  |
|  |  |  |  | -49 | -63 | -14 | < .001 | 4.52 |  |  |
|  |  |  |  | 48 | -55 | -18 | < .001 | 4.71 |  |  |
| Note. F = Female, M = Male, ^ = Activation survived family wise error correction (*p* < 0.05). * = exceeded threshold of *p* < .05 corrected at the cluster level. ** = exceeded the threshold of *p* < .001 uncorrected at the cluster level | | | | | | | | | | |

**Table S3.** Clusters of significant activity associated with BSE.

| **Anatomical Label** | **BA** | **MNI Coordinates** | | | **ALE** | ***p*-value** | ***z*-score** |
| --- | --- | --- | --- | --- | --- | --- | --- |
|  |  | **X** | **Y** | **Z** |  |  |  |
| R Inferior Frontal Junction | 9 | 52 | 10 | 30 | 0.024 | < .001 | 5.80 |
| R Middle Frontal Gyrus | 46 | 48 | 34 | 12 | 0.016 | < .001 | 4.34 |
| R Inferior Frontal Gyrus | 45 | 52 | 24 | 8 | 0.015 | < .001 | 4.15 |
| R Fusiform Body Area | 37 | 42 | -52 | -18 | 0.017 | < .001 | 4.64 |

Note. BA = Brodmann Area; MNI = Montreal Neurological Institute; R = Right hemisphere.

*Figure S1.* Significant clusters of activity from the ALE meta-analysis. Top image depicts a sagittal slice with one cluster in the right inferior frontal junction (IFJ), and one including the right middle frontal gyrus (MFG) and inferior frontal gyrus (IFG). Bottom image depicts an axial slice with one cluster in the right fusiform body area (FBA). Colour scale represents associated ALE statistic range from the analysis. R = right.

**References**

1.	Eickhoff SB, Nichols TE, Laird AR, Hoffstaedter F, Amunts K, Fox PT, et al. Behavior, sensitivity, and power of activation likelihood estimation characterized by massive empirical simulation. Neuroimage. 2016;137:70-85.

2.	Eickhoff SB, Bzdok D, Laird AR, Kurth F, Fox PT. Activation likelihood estimation meta-analysis revisited. Neuroimage. 2012;59(3):2349-61.

3.	Eickhoff SB, Laird AR, Grefkes C, Wang LE, Zilles K, Fox PT. Coordinate-based activation likelihood estimation meta-analysis of neuroimaging data: a random-effects approach based on empirical estimates of spatial uncertainty. Hum Brain Mapp. 2009;30(9):2907-26.

4.	Turkeltaub PE, Eden GF, Jones KM, Zeffiro TA. Meta-analysis of the functional neuroanatomy of single-word reading: method and validation. Neuroimage. 2002;16(3 Pt 1):765-80.

5.	Turkeltaub PE, Eickhoff SB, Laird AR, Fox M, Wiener M, Fox P. Minimizing within-experiment and within-group effects in Activation Likelihood Estimation meta-analyses. Hum Brain Mapp. 2012;33(1):1-13.

6.	Müller VI, Cieslik EC, Laird AR, Fox PT, Radua J, Mataix-Cols D, et al. Ten simple rules for neuroimaging meta-analysis. Neuroscience & Biobehavioral Reviews. 2018;84:151-61.
